# Supplementary material for: Caenorhabditis elegans SynMuv B gene activity is down-regulated during a viral infection to enhance RNA interference
Source: bioRxiv. 2024 Jul 16:2024.07.12.603258. Preprint. [Version 1] doi: 10.1101/2024.07.12.603258 (PMC11275910; doi:10.1101/2024.07.12.603258)

bioRxiv preprint doi: https://doi.org/10.1101/2024.07.12.603258; this version posted July 16, 2024. The copyright holder for this preprint (which was not certified by peer review) is the author/funder, who has granted bioRxiv a license to display the preprint in perpetuity. It is made available under aCC-BY 4.0 International license.

TABLE CAPTIONS

S1 Table: Top 50 upregulated Genes in *lin-15B(n744*), mutant in starved L1 animals. The 50 most highly upregulated genes in *lin-15(n744)* mutants during the L1 stage. The log2 Fold-change and the corresponding false discovery rate (FDR), value for each gene that is listed is shown. The gene expression data was derived from our analysis of mRNA seq data sets *[GSM4697084- N2 - Rep 1/L1, GSM4697085- N2 - Rep 2/L1 against GSM4697102- lin-15b(n744)- rep1/L1, GSM4697105- lin-15b(n744)- rep2/L1*], that were obtained from the NCBI Geo collection.

S2 Table: Top 50 upregulated Genes in *lin-35(n745*), mutant in starved L1 animals. The 50 most highly upregulated genes in *lin-35(n745)* mutants during the L1 stage. The log2 Fold-change and the corresponding false discovery rate (FDR), value for each gene that is listed is shown. The gene expression data was derived from our analysis of mRNA seq data sets *GSM4697089- lin-35[JA1507(n745) rep1/L1, GSM4697090- lin-35[JA1507(n745) rep1/L1],* that were obtained from the NCBI Geo collection.

bioRxiv preprint doi: https://doi.org/10.1101/2024.07.12.603258; this version posted July 16, 2024. The copyright holder for this preprint (which was not certified by peer review) is the author/funder, who has granted bioRxiv a license to display the preprint in perpetuity. It is made available under aCC-BY 4.0 International license.

S3 Table: Top 50 upregulated Genes in *lin-35(n745*), mutant at the L3 Stage. The top 50 most highly upregulated genes in *lin-35(n745)* mutants during the L3 stage. The log2 Fold-change and the corresponding false discovery rate (FDR), value for each gene that is listed is shown. [*GSM1534084- N2-rep1 L3, GSM1534085- N2-rep2 compared against GSM1534086- lin-35[JA1507(n745) rep1 L3 and GSM1534087- lin-35[JA1507(n745) rep1 L3],* that were obtained from the NCBI Geo collection.

FIGURE CAPTIONS

bioRxiv preprint doi: https://doi.org/10.1101/2024.07.12.603258; this version posted July 16, 2024. The copyright holder for this preprint (which was not certified by peer review) is the author/funder, who has granted bioRxiv a license to display the preprint in perpetuity. It is made available under aCC-BY 4.0 International license.

S1 Fig. Gene expression analysis reveals antiviral defense genes are upregulated in SynMuv B mutants. Volcano plots of a gene expression analysis done on mRNA seq data available through NCBI Geo for the null mutants of *lin-35(n745); glp-4(bn2)* and *glp-4(bn2); lin-15b(n744).* Highlighted are those GeneIDs belonging to genes that have been previously reported to play a role in antiviral defense response in worms. These genes are also listed on the right. Each of the highlighted GeneIDs have a cutoff of a logFC of 2.32 (i.e. a 5-fold change).

S2 Fig. Ectopic *lag-2p::GFP* expression in the intestinal cells upon virus-infection remains unaltered upon removal of *isw-1* activity by RNAi. (A). Fluorescent micrographs showing two *lag-2p::GFP* expressing animals that are infected with the Orsay virus raised on *E. coli* that express double stranded RNA against either empty vector (control) or the synMuv suppressor gene *isw-1*. Expression of *lag-2p::GFP* within the distal tip cells is indicated by yellow arrow heads and the ectopic fluorescence of *lag-2p::GFP* in the intestine is also indicated. (B). Quantification of the fluorescence intensity of *lag-2p::GFP* within the intestine of animals that are infected with the Orsay virus raised on *E. coli* that express double stranded RNA against either the L4440 empty vector (labelled as control) or *isw-1* is shown. A schematic of the general orientation of the worm is shown above the image panels. Scale bar is indicated.

bioRxiv preprint doi: https://doi.org/10.1101/2024.07.12.603258; this version posted July 16, 2024. The copyright holder for this preprint (which was not certified by peer review) is the author/funder, who has granted bioRxiv a license to display the preprint in perpetuity. It is made available under aCC-BY 4.0 International license.

S3 Fig. **Spatiotemporal expression pattern of ERGO-1 remains unperturbed by the loss of function of synMuv B genes**. (A-F). Brightfield and GFP channel micrographs depicting the temporal expression pattern of *ergo-1::GFP* from a transgenic strain that expresses a full length ERGO-1 PIWI protein fused at its C terminus to GFP . (G-R). *ergo-1::GFP* expression after synMuv B (*lin-9 or lin-13*), or Muv suppressor (*isw-1*) RNAi. Developmental stage is labelled. Scale bar is indicated.

S4 Fig. LIN-15B C-terminal protein fusion::EGFP localization in hypodermal and intestinal cells in *C. elegans*. (A-E). Brightfield and EGFP micrographs of LIN-15B localization in representative hypodermal (panels A & B), intestinal nuclei (panels C,D &E). Abbreviations for the different intestinal cells that are boxed is provided below the image panels. Scale bar is indicated.

S5 Fig. Quantitation of LIN-15B::EGFP fluorescence intensity and number of subnuclear foci. (A&B). Quantification of EGFP fluorescence and number of subnuclear LIN-15B foci in 3 representative hypodermal cells for synMuv B (*lin-9, lin-13, lin-35, lin-37 and lin-52*), Eri associated-gene (*eri-6*), synMuv suppressors (*mes-4, isw-1*), RNAi defective genes (*rde-1, rde-4, mut-16*) under RNAi conditions. (C&D). Quantification of EGFP fluorescence and number of subnuclear LIN-15B foci within 6 representative intestinal cells for synMuv B (*lin-9, lin-13, lin-35, lin-37 and lin-52*), Eri associated-gene (*eri-6*), synMuv suppressors (*mes-4, isw-1*), RNAi defective genes (*rde-1, rde-4, mut-16*) under RNAi conditions. In the graphs shown in C and D the abbreviations AD refers to anterior dorsal cell, AV refers to anterior ventral cell, MA refers to midgut anterior cell and MP refers to midgut posterior cell, PD refers to posterior gut dorsal cell and PV refers to posterior gut ventral cell.

bioRxiv preprint doi: https://doi.org/10.1101/2024.07.12.603258; this version posted July 16, 2024. The copyright holder for this preprint (which was not certified by peer review) is the author/funder, who has granted bioRxiv a license to display the preprint in perpetuity. It is made available under aCC-BY 4.0 International license.

S6 Fig. Quantification of LIN-15B::EGFP fusion protein fluorescence intensity and number of subnuclear foci in *lin-35(n745)* null mutants. (A&B). EGFP fluorescence and number of subnuclear LIN-15B foci within 3 representative hypodermal cells under RNAi conditions targeting L4440 vector (control), RNAi defective genes (*rde-1, mut-16*) and Muv suppressor (*isw-1 and mes-4*). (C&D). Intensity of EGFP fluorescence and number of subnuclear LIN-15B foci within 6 representative intestinal cells where the abbreviations AD refers to anterior dorsal cell, AV refers to anterior ventral cell, MA refers to midgut anterior cell and MP refers to midgut posterior cell, PD refers to posterior gut dorsal cell and PV refers to posterior gut ventral cell. RNAi was performed targeting L4440 vector (control), RNAi defective genes (*rde-1, mut-16*) and Muv suppressor genes (*isw-1 and mes-4*). These experiments were carried out in a *lin-35(n745)* null background.

S7 Fig. LIN-35::EGFP localization in the nucleolus of *C. elegans* intestinal cells. (A & B). Fluorescent micrographs of LIN-35::EGFP fusion protein localization in the intestinal nucleoli of wild-type animals. Magnification used to visualize the cells is indicated. The image shown in panel B are the same cells that are boxed in panel A, visualized under a higher magnification. (C). Fluorescent micrographs of LIN-35::EGFP localization within the intestinal cells (boxed in panel A), of the F1 progeny of worms raised on *E. coli* expressing double stranded RNA against either empty vector (l4440) or various synMuv B genes (*lin-9, lin-13, lin-15b, lin-37, lin-52, lin-54, lin-61, tam-1, hpl-1 and dpl-1*) or a synMuv A gene (*lin-8*) is shown. Arrow heads indicate the presence of a nucleolar inclusion of LIN-35::EGFP. (D). Quantification of the number of LIN-35::EGFP nucleolar inclusions that are seen in animals raised on *E. coli* expressing double stranded RNA against either empty vector (l4440) or various synMuv B genes (*lin-9, lin-13, lin-15b, lin-37, lin-52, lin-54, lin-61, tam-1, hpl-1 and dpl-1*) or a synMuv A gene (*lin-8*) is shown. (E). Quantification of the number of LIN-35::EGFP nucleolar inclusions that are seen in animals

bioRxiv preprint doi: https://doi.org/10.1101/2024.07.12.603258; this version posted July 16, 2024. The copyright holder for this preprint (which was not certified by peer review) is the author/funder, who has granted bioRxiv a license to display the preprint in perpetuity. It is made available under aCC-BY 4.0 International license.

raised on *E. coli* expressing double stranded RNA against either empty vector (l4440) or various genes that are critical for the worms ability to perform RNAi. Scale bar is indicated.

S8 Fig. *lin-15b(-)* animals exhibit altered intestinal nuclear morphology

Top panel shows wild-type *JJ2284* animals under no virus and Orsay virus-infected conditions. Lower panel shows a second independent line of *lin-15b(W485*)*; JJ2284 animals under no virus and Orsay virus-infected conditions. White arrows indicate elongated intestinal nuclei that are elongated. Scale bar is indicated.

S9 Fig. Comparisons of normalized reads of indicated classes of small RNAs in *lin-35(n745); glp-4(bn2)* with *glp-4(bn2)*. Dark grey data points represent small RNAs that are differentially expressed by 5-fold and adjusted p value < 0.05. Colored data points represent indicated classes of small RNAs. The rest of the small RNAs are represented as light grey data points. Lines denoting equal, 5-fold increased and 5-fold decreased expression are shown as light grey dashed lines. The numbers indicate the number of small RNAs upregulated in *lin-35(n745); glp-4(bn2)* (upper) and *glp-4(bn2)* (lower).

S10 Fig. Comparisons of normalized reads of indicated classes of small RNAs in *lin-15b(n744); glp-4(bn2)* with *glp-4(bn2)*. Dark grey data points represent small RNAs that are differentially expressed by 5-fold and adjusted p value < 0.05. Colored data points represent indicated classes of small RNAs. The rest of the small RNAs are represented as light grey data points. Lines denoting equal, 5-fold increased and 5-fold decreased expression are shown as light grey dashed lines. The numbers indicate the number of small RNAs upregulated in *lin-15b(n744); glp-4(bn2)* (upper) and *glp-4(bn2)* (lower).

bioRxiv preprint doi: https://doi.org/10.1101/2024.07.12.603258; this version posted July 16, 2024. The copyright holder for this preprint (which was not certified by peer review) is the author/funder, who has granted bioRxiv a license to display the preprint in perpetuity. It is made available under aCC-BY 4.0 International license.

S11 Fig. Comparisons of normalized reads of indicated classes of small RNAs in *lin-35(n745)* with wildtype. Dark grey data points represent small RNAs that are differentially expressed by 5-fold and adjusted p value < 0.05. Colored data points represent indicated classes of small RNAs. The rest of the small RNAs are represented as light grey data points. Lines denoting equal, 5-fold increased and 5-fold decreased expression are shown as light grey dashed lines. The numbers indicate the number of small RNAs upregulated in *lin-35(n745)* (upper) and wildtype (lower).

S12 Fig. Comparisons of normalized reads of indicated classes of small RNAs in *lin-15b(n744)* with wildtype. Dark grey data points represent small RNAs that are differentially expressed by 5-fold and adjusted p value < 0.05. Colored data points represent indicated classes of small RNAs. The rest of the small RNAs are represented as light grey data points. Lines denoting equal, 5-fold increased and 5-fold decreased expression are shown as light grey dashed lines. The numbers indicate the number of small RNAs upregulated in *lin-15b(n744)*(upper) and wildtype (lower).

S13 Fig. Comparisons of normalized reads of indicated classes of small RNAs in *lin-9(n112)* with wildtype. B Dark grey data points represent small RNAs that are differentially expressed by 5-fold and adjusted p value < 0.05. Colored data points represent indicated classes of small RNAs. The rest of the small RNAs are represented as light grey data points. Lines denoting equal, 5-fold increased and 5-fold decreased expression are shown as light grey dashed lines. The numbers indicate the number of small RNAs upregulated in *lin-9(n112)* (upper) and wildtype (lower).

S14 Fig. Comparisons of normalized reads of indicated classes of small RNAs in *lin-52(n771)* with wildtype. Dark grey data points represent small RNAs that are differentially

bioRxiv preprint doi: https://doi.org/10.1101/2024.07.12.603258; this version posted July 16, 2024. The copyright holder for this preprint (which was not certified by peer review) is the author/funder, who has granted bioRxiv a license to display the preprint in perpetuity. It is made available under aCC-BY 4.0 International license.

expressed by 5-fold and adjusted p value < 0.05. Colored data points represent indicated classes of small RNAs. The rest of the small RNAs are represented as light grey data points. Lines denoting equal, 5-fold increased and 5-fold decreased expression are shown as light grey dashed lines. The numbers indicate the number of small RNAs upregulated in *lin-52(n771)*(upper) and wildtype (lower).

ADDITIONAL SUPPORTING INFORMATION DATA FILES: [uploaded separately]

Additional supporting information data table 1. List of genes to which differentially regulated small RNAs map in *lin-35(n745); glp-4(bn2)* and *lin-15b(n744); glp-4(bn2)* in comparison with *glp-4(bn2).* The siRNAs that are either Up or Down by a minimum factor of a 5-fold difference in the *lin-15(n744)* and *lin-35(n745)* mutant backgrounds are presented. See the labeled tabs for the genotype information.

Additional supporting information data table 2. Gene expression changes in *lin-35(n745)* starved L1 animals. Shown here are the list of significant gene expression changes via mRNA seq that are observed in the *lin-35(n745)* synMuv B mutant animals at the L1 stage. The data shown here was obtained from the NCBI geo collection, *GSM4697089 [lin-35(n745) starved L1-rep1)], GSM4697090 (lin-35(n745) starved L1-rep2)].*

Additional supporting information data table 3. Gene expression changes in *lin-35(n745)* starved L3-stage animals. Shown here are the list of significant gene expression changes via mRNA seq that are observed in the *lin-35(n745)* synMuv B mutant animals at the L3-stage. The data shown here was obtained from the NCBI geo collection, *GSM1534086 (lin-35[JA1507(n745) L3-rep1), GSM1534087(lin-35[JA1507(n745) L3-rep2)].*

bioRxiv preprint doi: https://doi.org/10.1101/2024.07.12.603258; this version posted July 16, 2024. The copyright holder for this preprint (which was not certified by peer review) is the author/funder, who has granted bioRxiv a license to display the preprint in perpetuity. It is made available under aCC-BY 4.0 International license.

Additional supporting information data table 4. Gene expression changes in *lin-15(n744)* starved L1-stage animals. Shown here are the list of significant gene expression changes via mRNA seq that are observed in the *lin-15(n744)* synMuv B mutant animals at the L1-stage. The data shown here was obtained from the NCBI geo collection, *GSM4697102 (lin-15B(n744) starved L1-rep1), GSM4697105 (lin-15B(n744) starved L1-rep2)].*

bioRxiv preprint doi: https://doi.org/10.1101/2024.07.12.603258; this version posted July 16, 2024. The copyright holder for this preprint (which was not certified by peer review) is the author/funder, who has granted bioRxiv a license to display the preprint in perpetuity. It is made available under aCC-BY 4.0 International license.

Gene	log2 fold change	FDR
pals-5	9.06	5.80E-07
pals-11	8.66	1.51E-05
pals-12	8.01	1.01E-06
F15H10.9	7.84	0.00602431
pals-10	7.78	0.0035545
Y105C5A.1269	7.63	3.18E-06
H05C05.3	7.54	4.39E-05
F15H10.12	7.16	0.00781933
Y82E9BL.19	7.06	2.88E-05
pals-29	6.99	1.63E-05
fbxa-75	6.97	2.01E-05
F15H10.10	6.72	0.00014014
F15H10.6	6.59	9.49E-05
C08E3.15	6.44	0.01574134
pes-2.1	6.43	0.28750516
pals-9	6.36	7.43E-05
Y54H5A.5	6.25	8.87E-09
F57G4.11	6.18	2.06E-05
Y45F10C.6	6.10	2.64E-05
Y61B8B.2	6.05	0.00502144
ZC434.8	5.84	3.27E-09
math-1	5.55	0.00161985
srw-85	5.51	1.43E-07
cpg-2	5.44	6.18E-07
Y51H7C.12	5.37	0.03414094
F37D6.3	5.37	4.15E-08
sdz-6	5.26	0.05818951
Y38E10A.3	5.26	0.19355176
F40G12.11	5.25	0.17805195
ZK250.14	5.25	0.00148968
Y57G7A.2	5.24	0.20756677
skr-9	5.19	0.11960343
sri-68	5.15	0.00162513
C08F11.7	5.14	0.00026807
F22E5.20	5.14	0.31126046
pals-3	5.11	0.00014288
Y39H10B.3	5.07	1.77E-06
F20E11.17	5.04	0.00089112
math-5	5.03	0.00911578
pals-28	4.99	0.00452157
fbxb-90	4.97	0.27388713
ZK666.11	4.94	0.03078516
C29A12.1	4.92	9.83E-08
T05A8.2	4.78	0.01574134
E02H9.3	4.75	9.83E-08
ucr-2.3	4.74	8.87E-09
C38D4.1	4.72	5.09E-07
F40E12.2	4.71	0.00044008
srg-53	4.70	0.0021712
pals-4	4.67	0.00050884
pals-14	4.67	2.95E-06

S1 Table*:* **Top 50 Upregulated Genes in *lin-15B(n744)*, mutant in starved L1 animals**

bioRxiv preprint doi: https://doi.org/10.1101/2024.07.12.603258; this version posted July 16, 2024. The copyright holder for this preprint (which was not certified by peer review) is the author/funder, who has granted bioRxiv a license to display the preprint in perpetuity. It is made available under aCC-BY 4.0 International license.

Gene	log2 fold change	FDR
T22C1.12	8.72	5.59E-05
pals-11	8.50	3.79E-06
fbxb-68	8.27	1.63E-05
F15H10.9	7.98	0.00124449
pals-5	7.59	8.19E-07
Y105C5A.1269	7.06	1.79E-06
pals-10	7.02	0.00195773
pals-29	6.92	3.66E-06
inx-22	6.90	1.11E-06
F15H10.12	6.71	0.00312932
fbxa-75	6.70	6.13E-06
pals-12	6.64	1.60E-06
F57G4.11	6.58	2.03E-06
H05C05.3	6.52	3.61E-05
F15H10.10	6.46	3.82E-05
F40E12.2	6.31	3.30E-06
srd-4	6.14	3.44E-06
sdz-6	6.12	0.00513928
srr-10	6.11	1.35E-05
F15H10.6	6.06	4.05E-05
pals-9	6.06	2.08E-05
F35E12.13	6.06	0.00110475
ZK250.14	6.03	5.42E-05
F47B8.14	6.02	0.05840114
C08E3.15	5.84	0.00745415
Y82E9BL.19	5.77	4.03E-05
ZK250.2	5.68	4.20E-05
ZK892.6	5.67	5.81E-06
Y57G7A.2	5.66	0.05702741
Y105C5B.20	5.65	0.00013254
pals-4	5.60	1.54E-05
hch-1	5.57	0.06142148
srh-16	5.54	9.34E-07
Y61B8B.2	5.46	0.00264392
F35G2.3	5.45	0.00027295
pals-3	5.45	1.40E-05
F08F3.8	5.44	3.24E-07
F22E5.20	5.40	0.12336736
Y38E10A.3	5.36	0.06585484
pes-2.1	5.36	0.21682155
C29A12.1	5.35	2.16E-08
C46C11.4	5.31	0.04733931
oac-32	5.27	8.90E-05
math-5	5.26	0.00111717
ZK250.15	5.25	0.00010885
ugt-26	5.24	1.23E-06
Y71G12A.4	5.23	1.60E-06
srw-85	5.23	7.14E-08
T24D5.5	5.21	0.00042663
F20E11.17	5.15	0.00013251
C25A11.1	5.15	5.61E-06

S2 Table*:* **Top 50 Upregulated Genes in *lin-35(n745)* mutant in starved L1 animals**

bioRxiv preprint doi: https://doi.org/10.1101/2024.07.12.603258; this version posted July 16, 2024. The copyright holder for this preprint (which was not certified by peer review) is the author/funder, who has granted bioRxiv a license to display the preprint in perpetuity. It is made available under aCC-BY 4.0 International license.

Gene	log2 fold change	FDR
fbxa-75	11.55	4.28E-04
pals-3	11.46	3.47E-04
pals-8	10.93	6.37E-04
pals-11	9.69	1.15E-03
F26F2.4	9.47	5.38E-03
F15H10.9	9.32	1.44E-02
pals-29	9.10	4.99E-04
oac-56	8.82	2.03E-02
B0507.15	8.81	4.53E-03
F40E12.2	8.79	5.73E-04
sdz-6	8.72	1.27E-02
fbxa-165	8.68	2.21E-04
F26F2.5	8.59	2.10E-03
F15H10.5	8.51	3.41E-02
srd-4	8.38	4.62E-04
E03H4.9	8.33	2.15E-03
Y54G2A.13	8.32	6.55E-03
srh-16	8.30	1.20E-04
K06A5.2	8.14	4.63E-02
F57G4.1	8.11	1.01E-02
C43D7.4	7.96	1.89E-02
F57G4.11	7.89	1.63E-04
pals-5	7.89	3.47E-04
Y39H10B.3	7.84	7.04E-05
fbxa-7	7.77	1.17E-03
math-16	7.77	7.70E-02
C04B4.2	7.75	1.04E-01
F45E4.6	7.74	8.46E-03
pals-9	7.72	9.99E-04
T27E7.9	7.67	3.72E-02
T24A6.7	7.63	2.10E-02
pals-7	7.51	2.97E-03
F37D6.3	7.48	9.45E-06
H04D03.6	7.43	3.21E-02
B0281.8	7.43	2.53E-02
pals-2	7.38	7.04E-05
F55G11.3	7.34	4.63E-02
math-17	7.34	4.79E-02
F15H10.6	7.33	1.45E-03
F26F2.3	7.13	2.69E-05
fbxb-68	7.10	2.79E-02
F36H5.10	7.09	1.55E-02
F35G2.3	7.06	1.01E-02
srg-31	7.04	2.75E-03
sri-68	7.03	3.12E-02
pals-33	6.97	9.45E-06
pals-4	6.89	1.15E-03
fbxa-173	6.85	1.42E-01
Y61B8B.2	6.82	1.83E-02
F20E11.17	6.79	2.83E-03

S3 Table*:* **Top 50 Upregulated Genes in *lin-35(n745)* mutant in L3 stage animals**

bioRxiv preprint doi: https://doi.org/10.1101/2024.07.12.603258; this version posted July 16, 2024. The copyright holder for this preprint (which was not certified by peer review) is the author/funder, who has granted bioRxiv a license to display the preprint in perpetuity. It is made available under aCC-BY 4.0 International license.

S1 Fig. Gene expression analysis reveals antiviral defense genes are upregulated in SynMuv B mutants

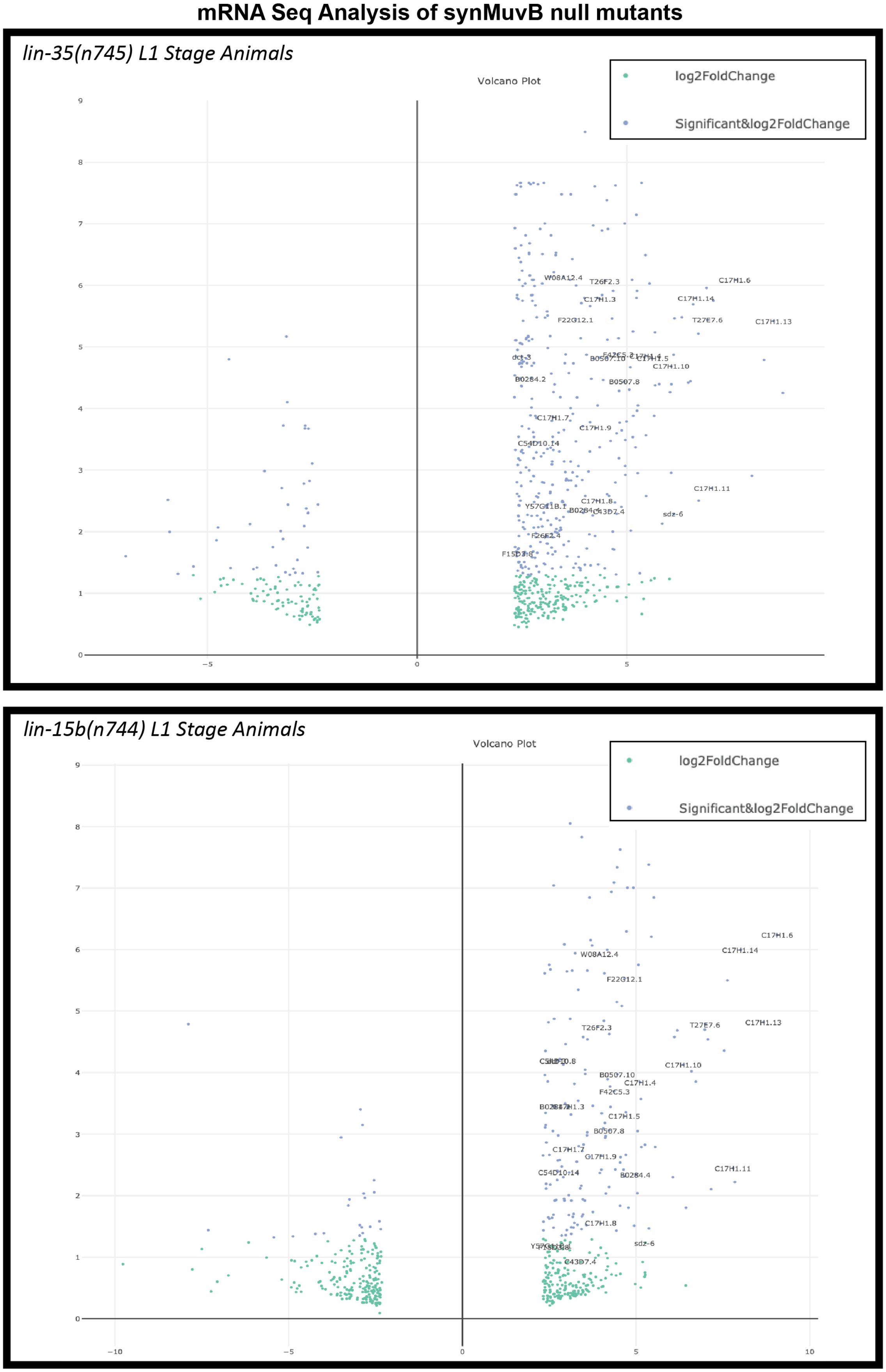

Genes highlighted:

pals-1/F15D3.8
pals-2/C17H1.3
pals-3/C17H1.4
pals-4/ C17H1.5
pals-5/C17H1.6
pals-6/C17H1.7
pals-7/C17H1.8
pals-8/C17H1.9
pals-9/C17H1.10
pals-10/C17H1.11
pals-11/C17H1.13
pals-12/ C17H1.14
pals-14/F22G12.1
pals-27/B0284.2
pals-28/B0284.4
pals-29/T27E7.6
pals-30/Y57G11B.1
pals-33/W08A12.4
pals-37/C54D10.14
B0507.10
B0507.8
F26F2.4
sdz-6
C43D7.4
F42C5.3
eol-1/T26F2.3

bioRxiv preprint doi: https://doi.org/10.1101/2024.07.12.603258; this version posted July 16, 2024. The copyright holder for this preprint (which was not certified by peer review) is the author/funder, who has granted bioRxiv a license to display the preprint in perpetuity. It is made available under aCC-BY 4.0 International license.

S2 Fig. Ectopic *lag-2p::GFP* expression in the intestinal cells upon virus-infection remains unaltered upon removal of *isw-1* activity by RNAi

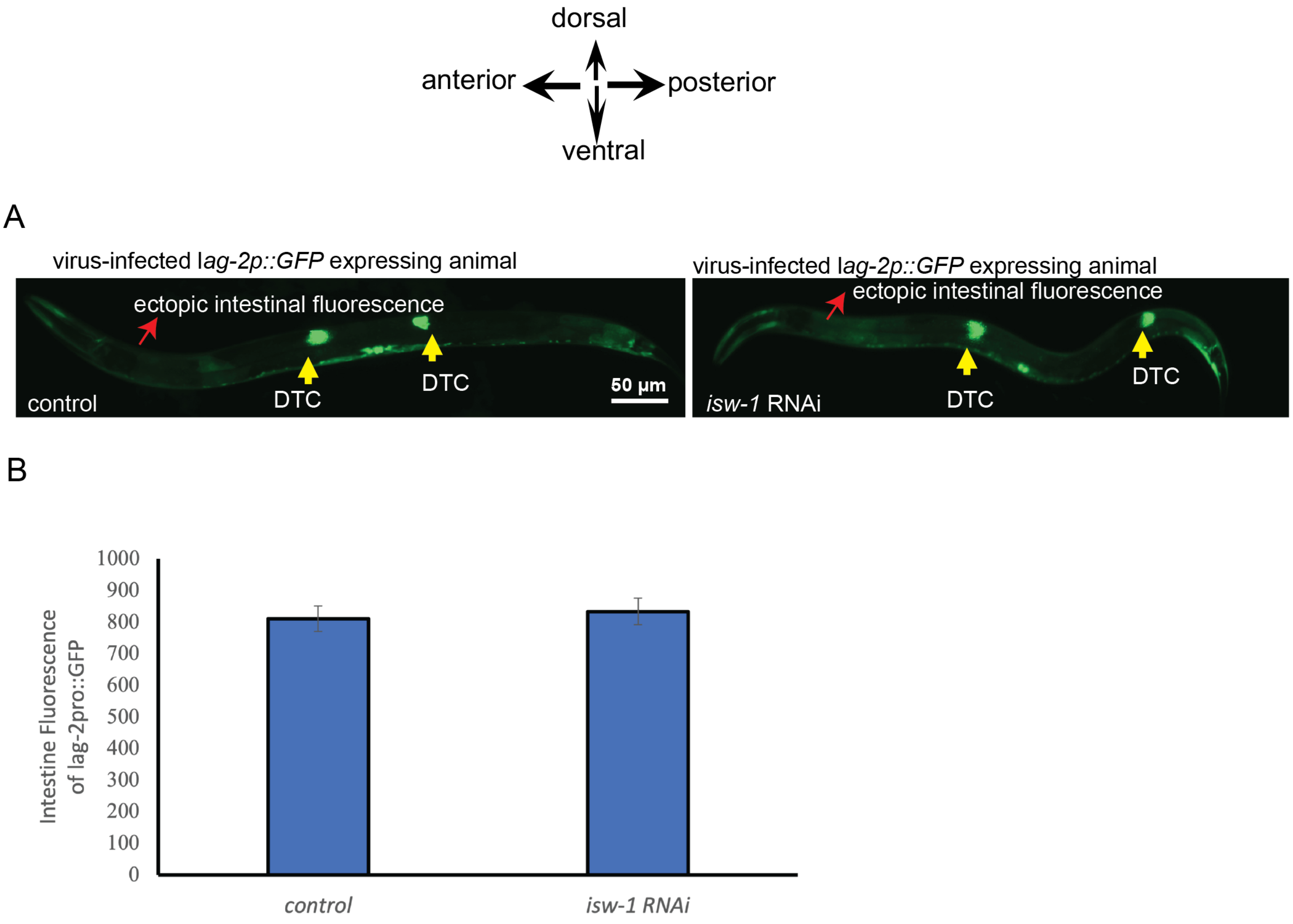

bioRxiv preprint doi: https://doi.org/10.1101/2024.07.12.603258; this version posted July 16, 2024. The copyright holder for this preprint (which was not certified by peer review) is the author/funder, who has granted bioRxiv a license to display the preprint in perpetuity. It is made available under aCC-BY 4.0 International license.

S3 Fig. Spatiotemporal expression pattern of ERGO-1 remains unperturbed by the loss of function of synMuv B genes

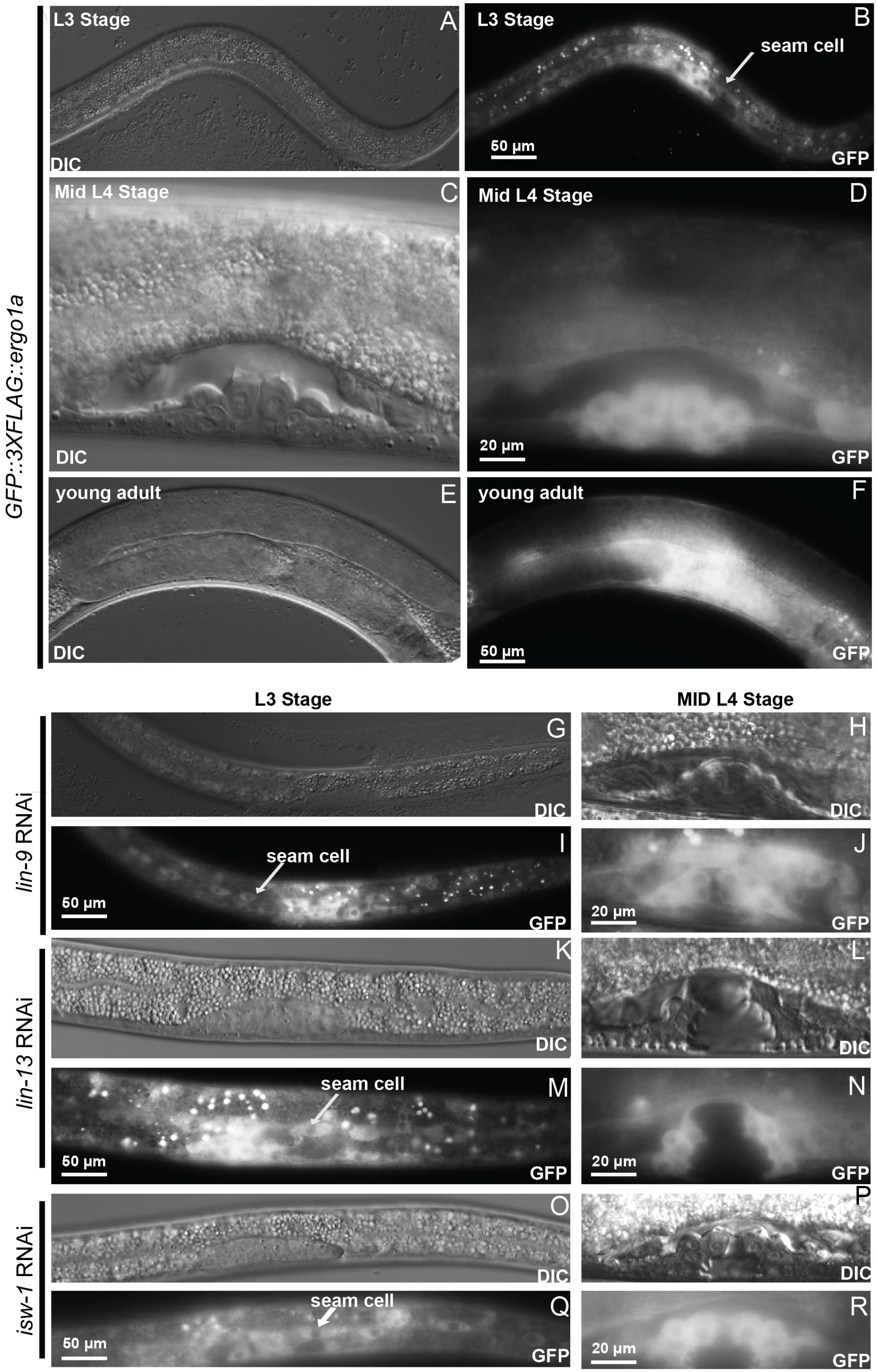

bioRxiv preprint doi: https://doi.org/10.1101/2024.07.12.603258; this version posted July 16, 2024. The copyright holder for this preprint (which was not certified by peer review) is the author/funder, who has granted bioRxiv a license to display the preprint in perpetuity. It is made available under aCC-BY 4.0 International license.

S4 Fig. LIN-15B C-terminal protein fusion::EGFP localization in hypodermal and intestinal cells in *C. elegans*

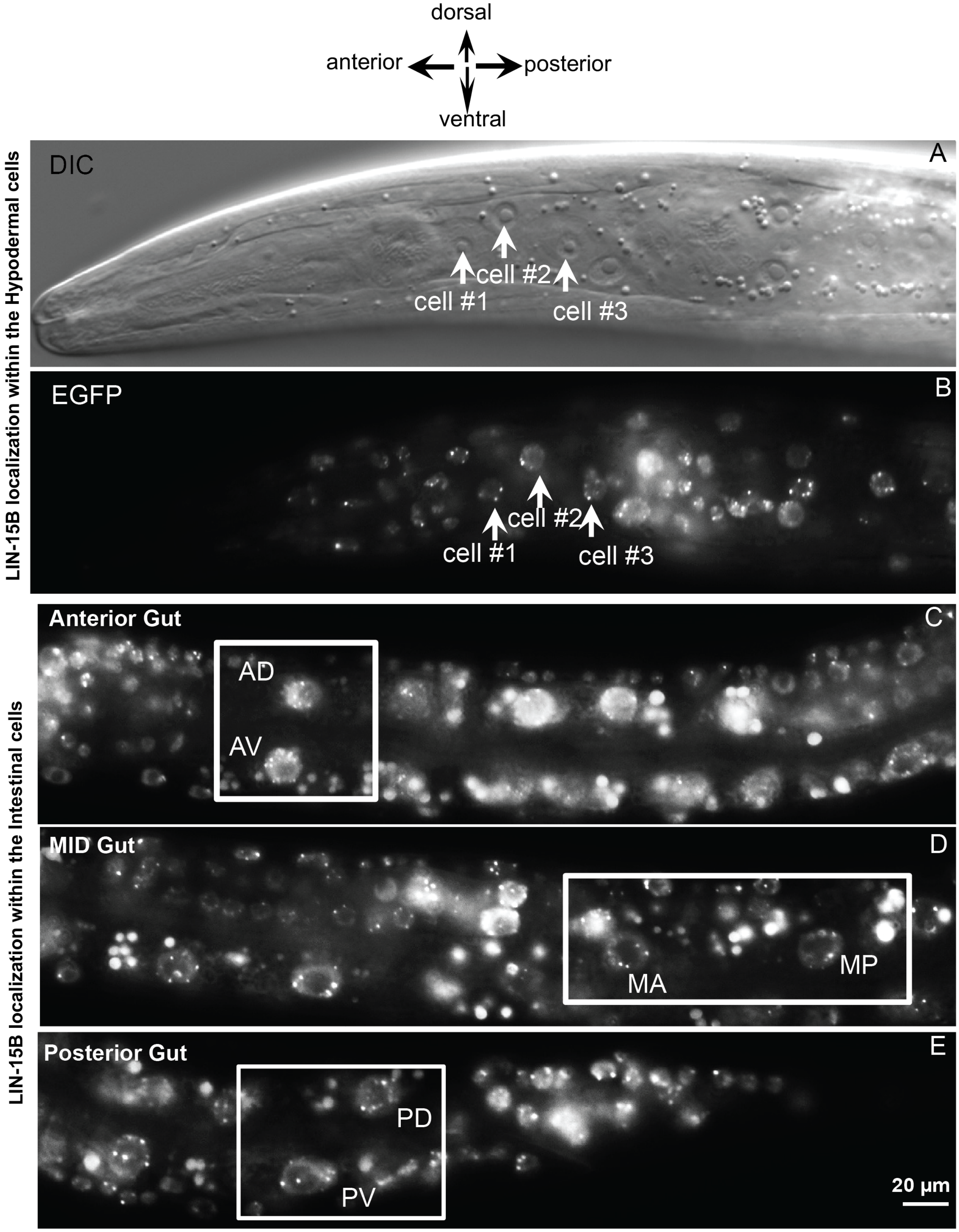

Strain: ***OP184 (unc-119(tm4063) III; wgls184)***
wgls184: [lin-15B::TY1::EGFP::3xFLAG + unc-119(+)]. TY1::EGFP::3xFLAG]

AD: anterior dorsal AV: anterior ventral MA: Midgut anterioir MP: Midgut posterioir
PD: posterior dorsal PV: posterioir ventral

bioRxiv preprint doi: https://doi.org/10.1101/2024.07.12.603258; this version posted July 16, 2024. The copyright holder for this preprint (which was not certified by peer review) is the author/funder, who has granted bioRxiv a license to display the preprint in perpetuity. It is made available under aCC-BY 4.0 International license.

S5 Fig. Quantitation of LIN-15B::EGFP fluorescence intensity and number of subnuclear foci

A

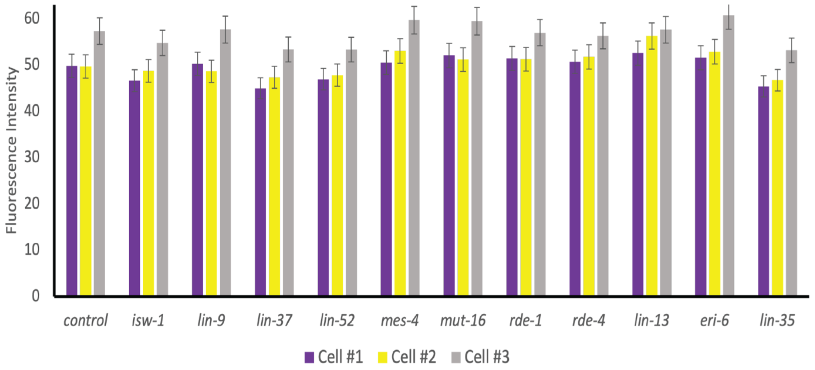

B

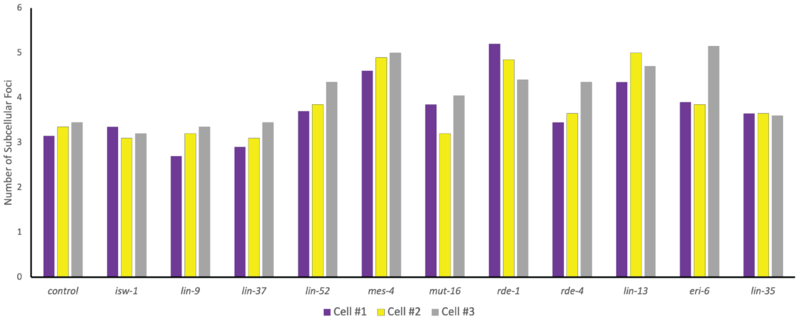

C

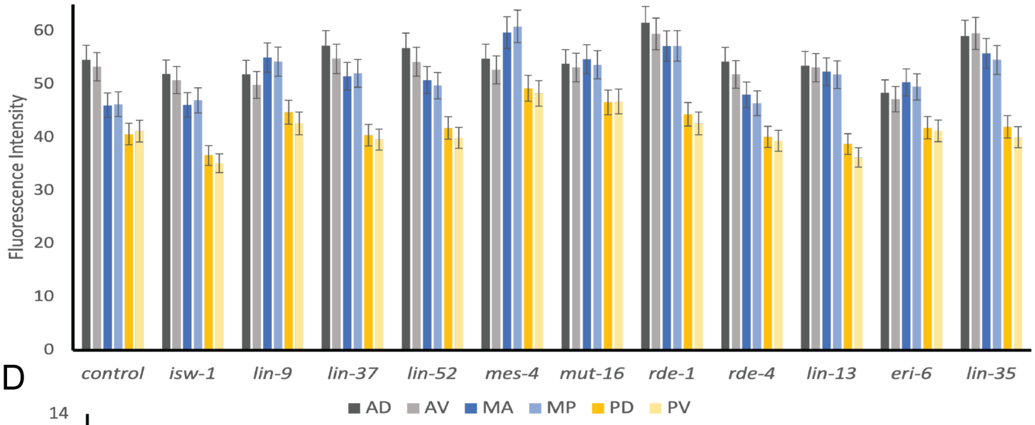

D

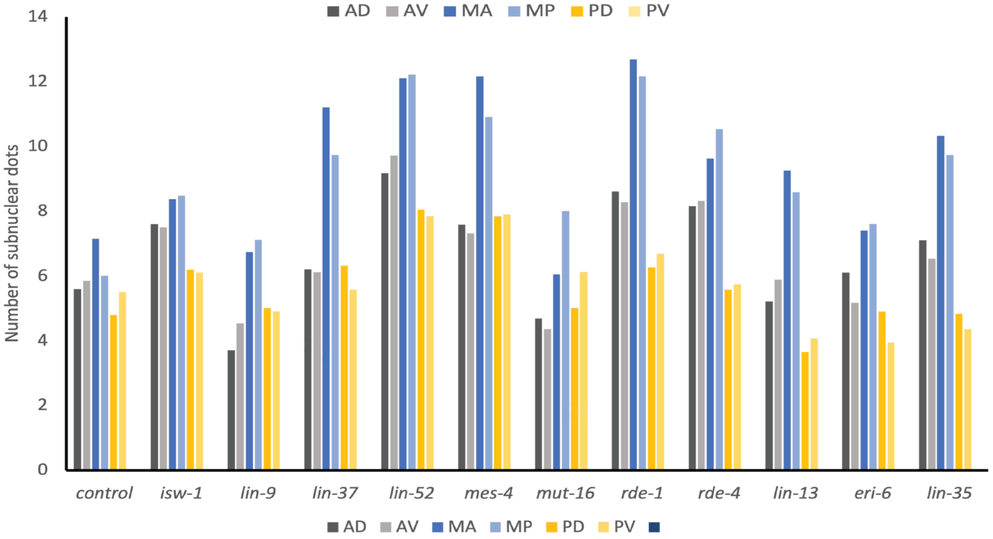

bioRxiv preprint doi: https://doi.org/10.1101/2024.07.12.603258; this version posted July 16, 2024. The copyright holder for this preprint (which was not certified by peer review) is the author/funder, who has granted bioRxiv a license to display the preprint in perpetuity. It is made available under aCC-BY 4.0 International license.

S6 Fig. Quantification of LIN-15B::EGFP fusion protein fluorescence intensity and number of subnuclear foci in *lin-35(n745)* null mutants

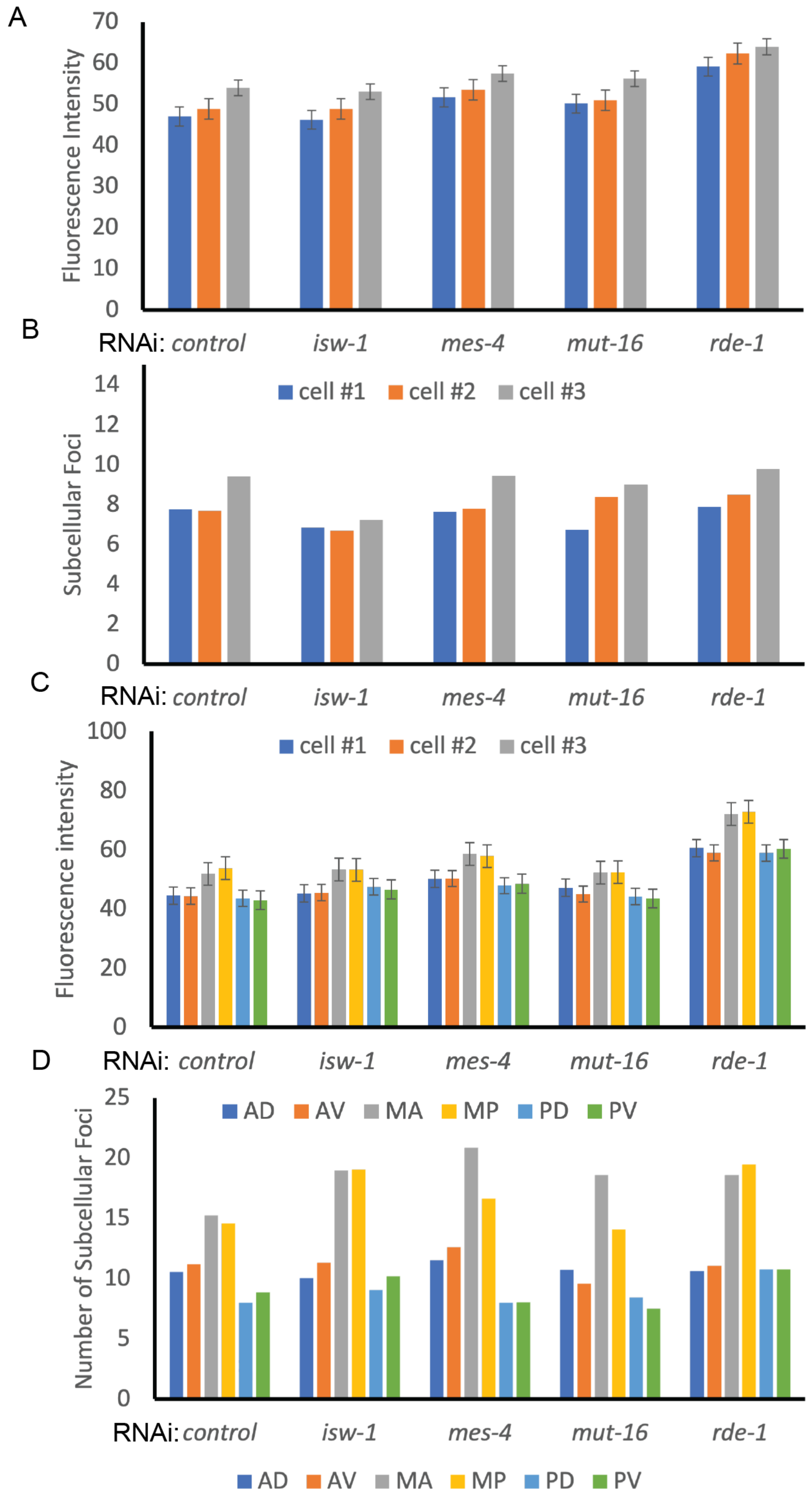

*All experiments carried out in a *lin-35(n745); LIN-15B::EGFP* background

bioRxiv preprint doi: https://doi.org/10.1101/2024.07.12.603258; this version posted July 16, 2024. The copyright holder for this preprint (which was not certified by peer review) is the author/funder, who has granted bioRxiv a license to display the preprint in perpetuity. It is made available under aCC-BY 4.0 International license.

S7 Fig. LIN-35::EGFP localization in the nucleolus of *C. elegans* intestinal cells

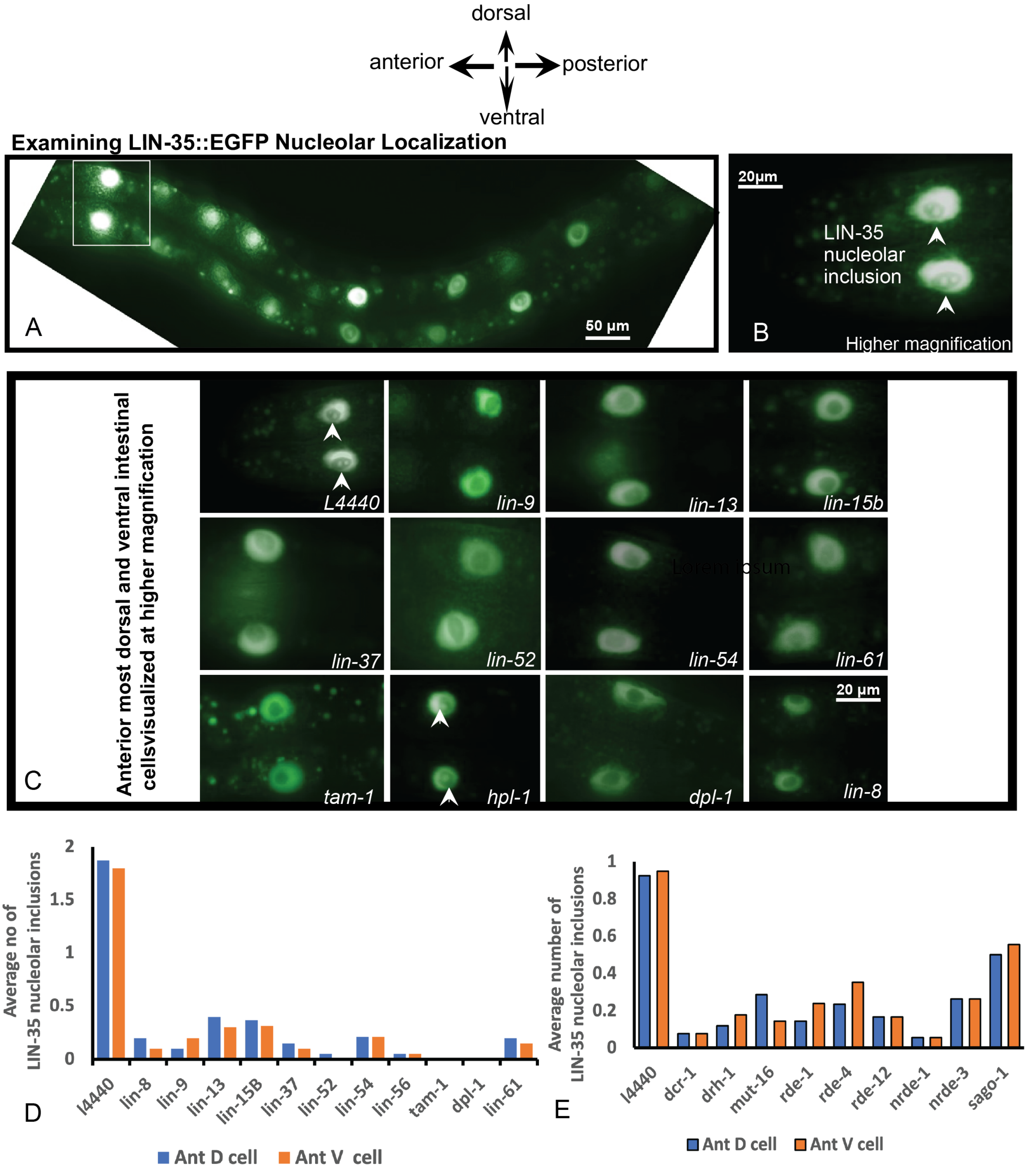

bioRxiv preprint doi: https://doi.org/10.1101/2024.07.12.603258; this version posted July 16, 2024. The copyright holder for this preprint (which was not certified by peer review) is the author/funder, who has granted bioRxiv a license to display the preprint in perpetuity. It is made available under aCC-BY 4.0 International license.

S8 Fig. *lin-15b(-)* animals exhibit altered intestinal nuclear morphology

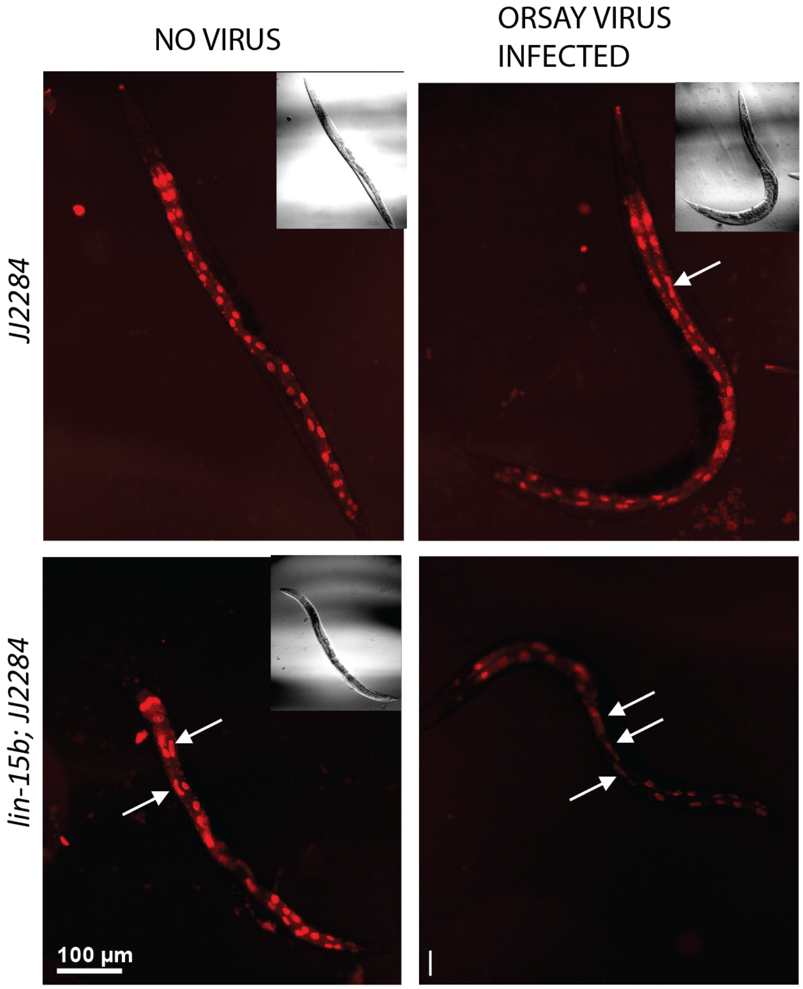

bioRxiv preprint doi: https://doi.org/10.1101/2024.07.12.603258; this version posted July 16, 2024. The copyright holder for this preprint (which was not certified by peer review) is the author/funder, who has granted bioRxiv a license to display the preprint in perpetuity. It is made available under aCC-BY 4.0 International license.

S9 Fig. Comparisons of normalized reads of indicated classes of small RNAs in *lin-35(n745); glp-4(bn2)* with *glp-4(bn2)*

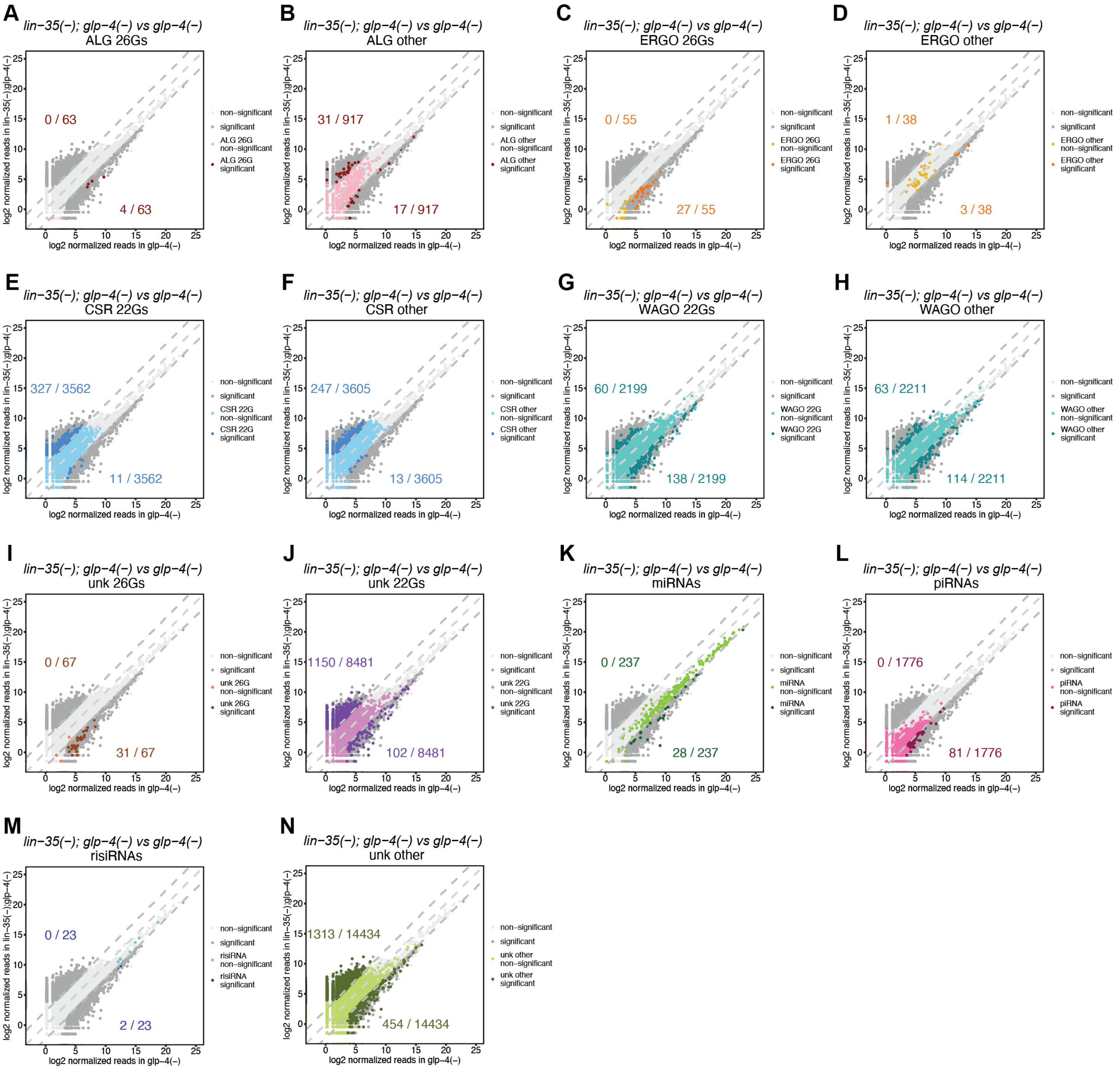

bioRxiv preprint doi: https://doi.org/10.1101/2024.07.12.603258; this version posted July 16, 2024. The copyright holder for this preprint (which was not certified by peer review) is the author/funder, who has granted bioRxiv a license to display the preprint in perpetuity. It is made available under aCC-BY 4.0 International license.

S10 Fig. Comparisons of normalized reads of indicated classes of small RNAs in *lin-15b(n744); glp-4(bn2)* with *glp-4(bn2)*

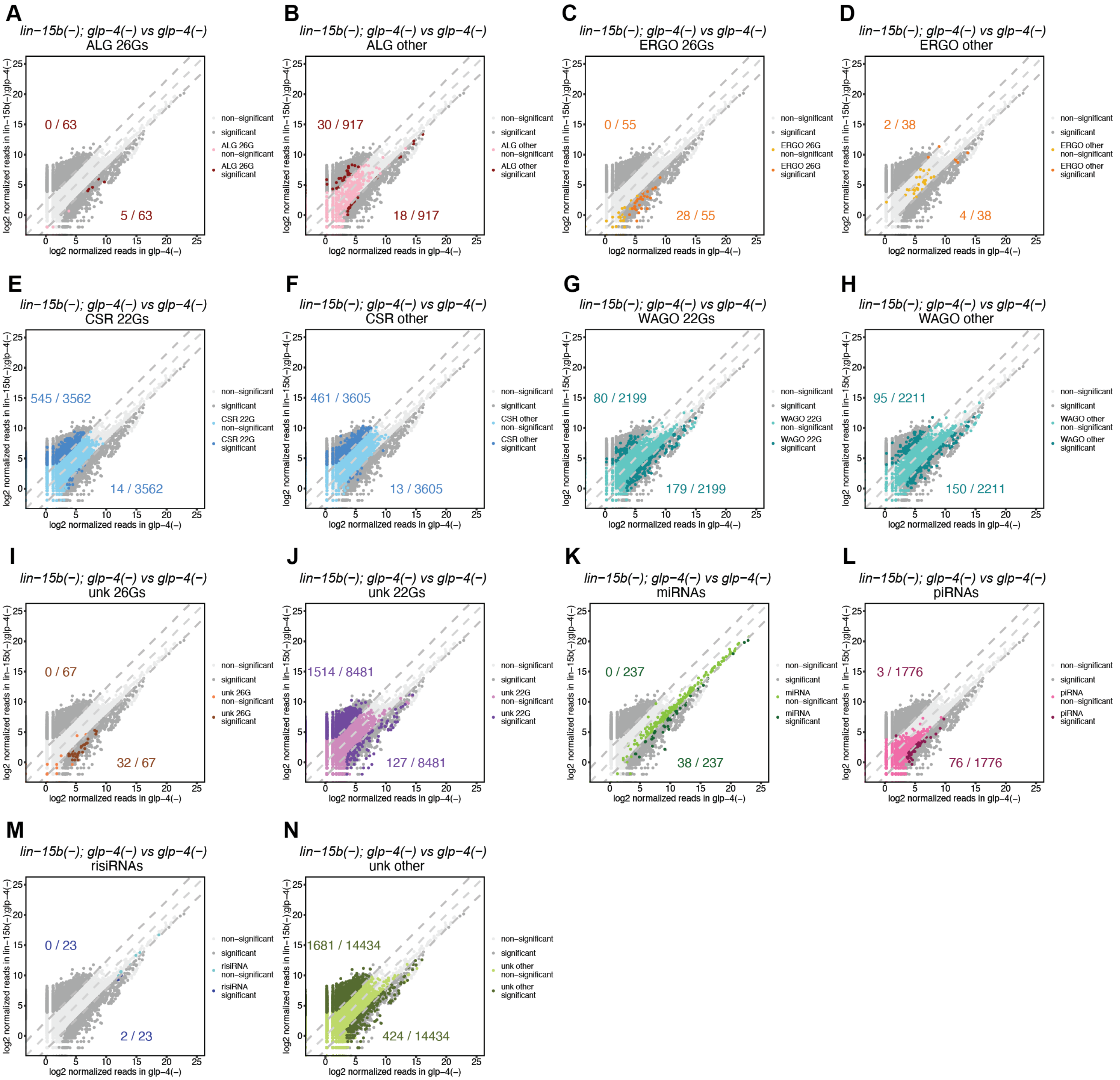

bioRxiv preprint doi: https://doi.org/10.1101/2024.07.12.603258; this version posted July 16, 2024. The copyright holder for this preprint (which was not certified by peer review) is the author/funder, who has granted bioRxiv a license to display the preprint in perpetuity. It is made available under aCC-BY 4.0 International license.

S11 Fig. Comparisons of normalized reads of indicated classes of small RNAs in *lin-35(n745)* with wildtype

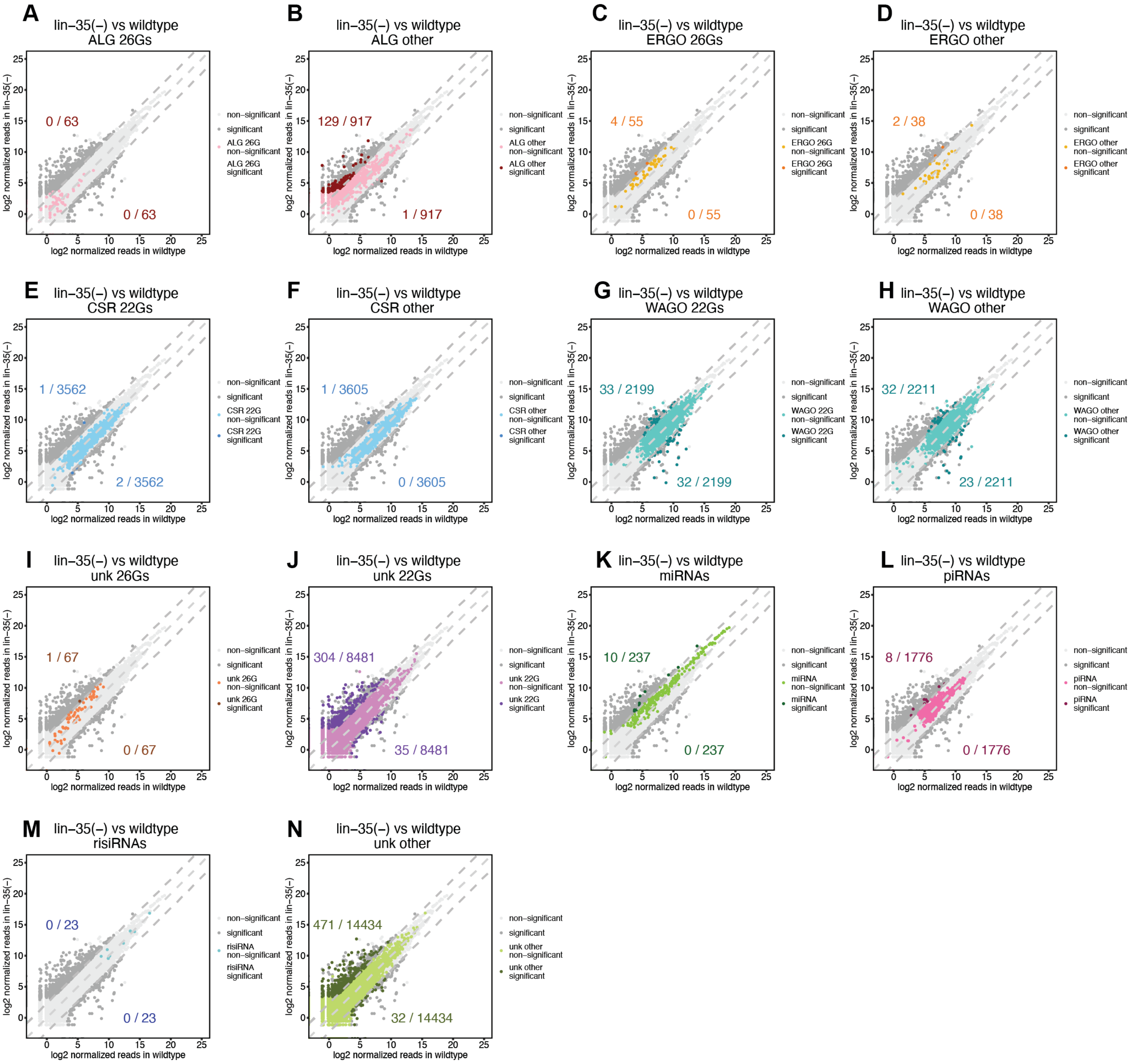

bioRxiv preprint doi: https://doi.org/10.1101/2024.07.12.603258; this version posted July 16, 2024. The copyright holder for this preprint (which was not certified by peer review) is the author/funder, who has granted bioRxiv a license to display the preprint in perpetuity. It is made available under aCC-BY 4.0 International license.

S12 Fig. Comparisons of normalized reads of indicated classes of small RNAs in *lin-15b(n744)* with wildtype

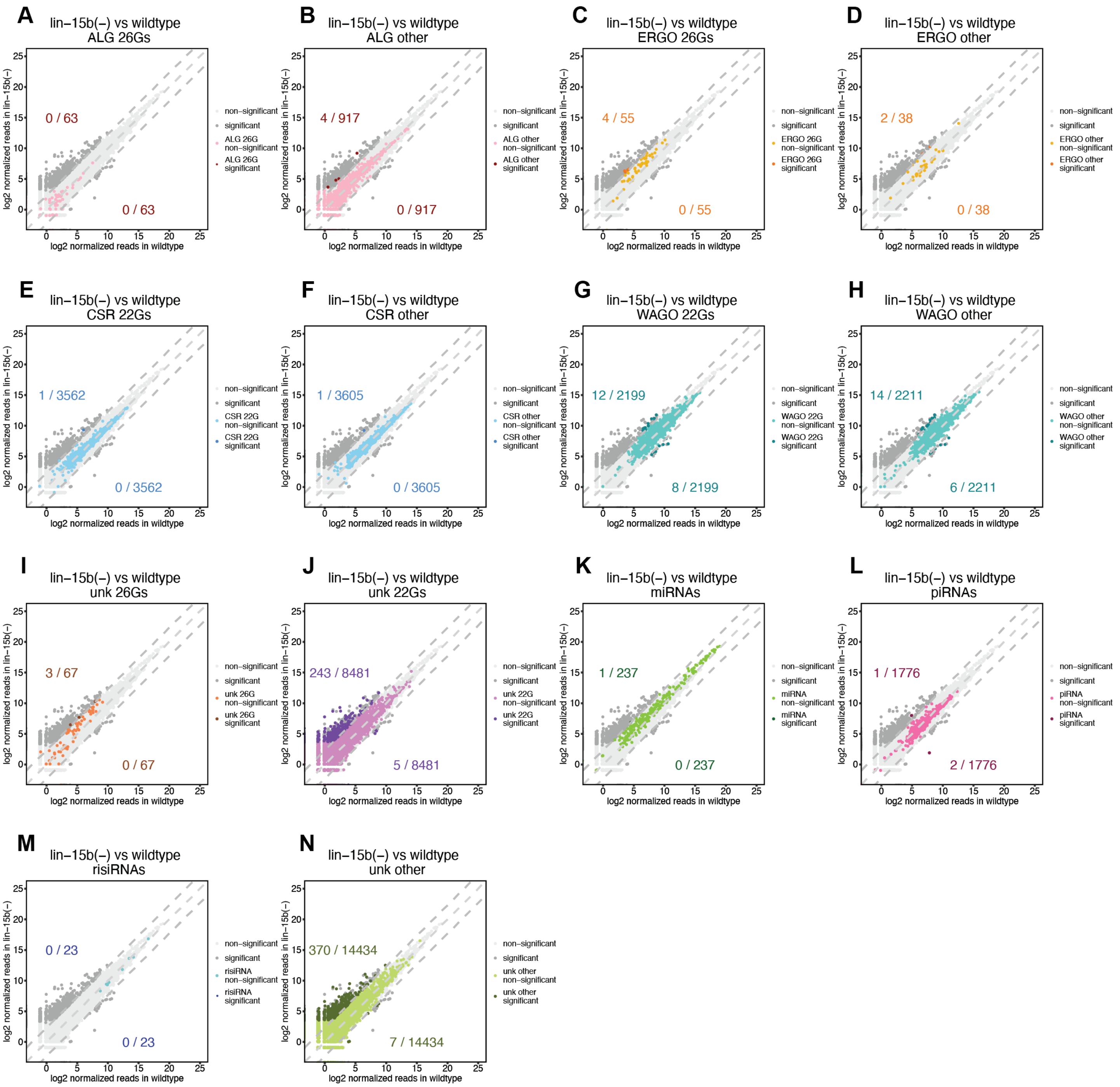

bioRxiv preprint doi: https://doi.org/10.1101/2024.07.12.603258; this version posted July 16, 2024. The copyright holder for this preprint (which was not certified by peer review) is the author/funder, who has granted bioRxiv a license to display the preprint in perpetuity. It is made available under aCC-BY 4.0 International license.

S13 Fig. Comparisons of normalized reads of indicated classes of small RNAs in *lin-9(n112)* with wildtype

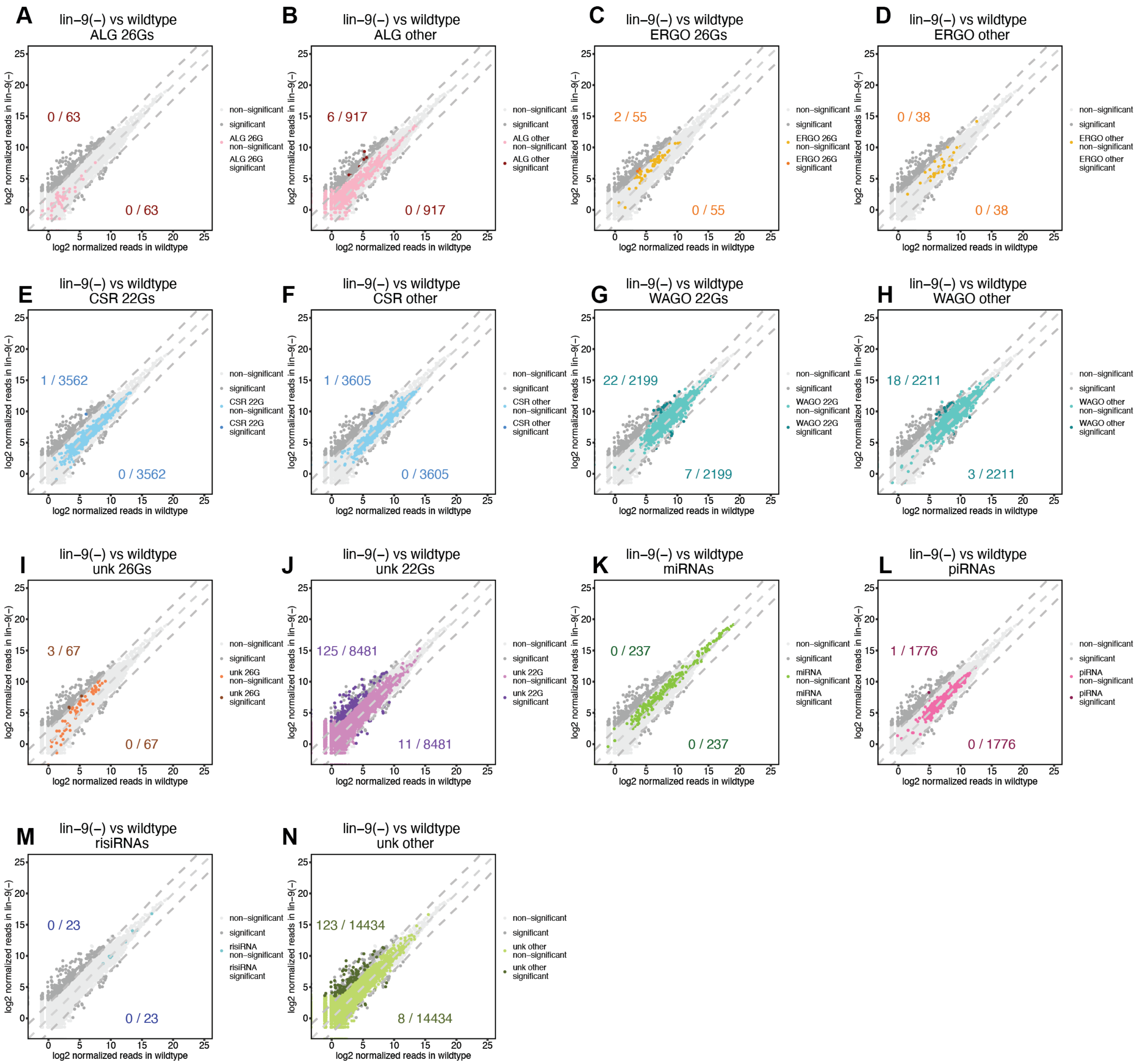

bioRxiv preprint doi: https://doi.org/10.1101/2024.07.12.603258; this version posted July 16, 2024. The copyright holder for this preprint (which was not certified by peer review) is the author/funder, who has granted bioRxiv a license to display the preprint in perpetuity. It is made available under aCC-BY 4.0 International license.

S14 Fig. Comparisons of normalized reads of indicated classes of small RNAs in *lin-52(n771)* with wildtype

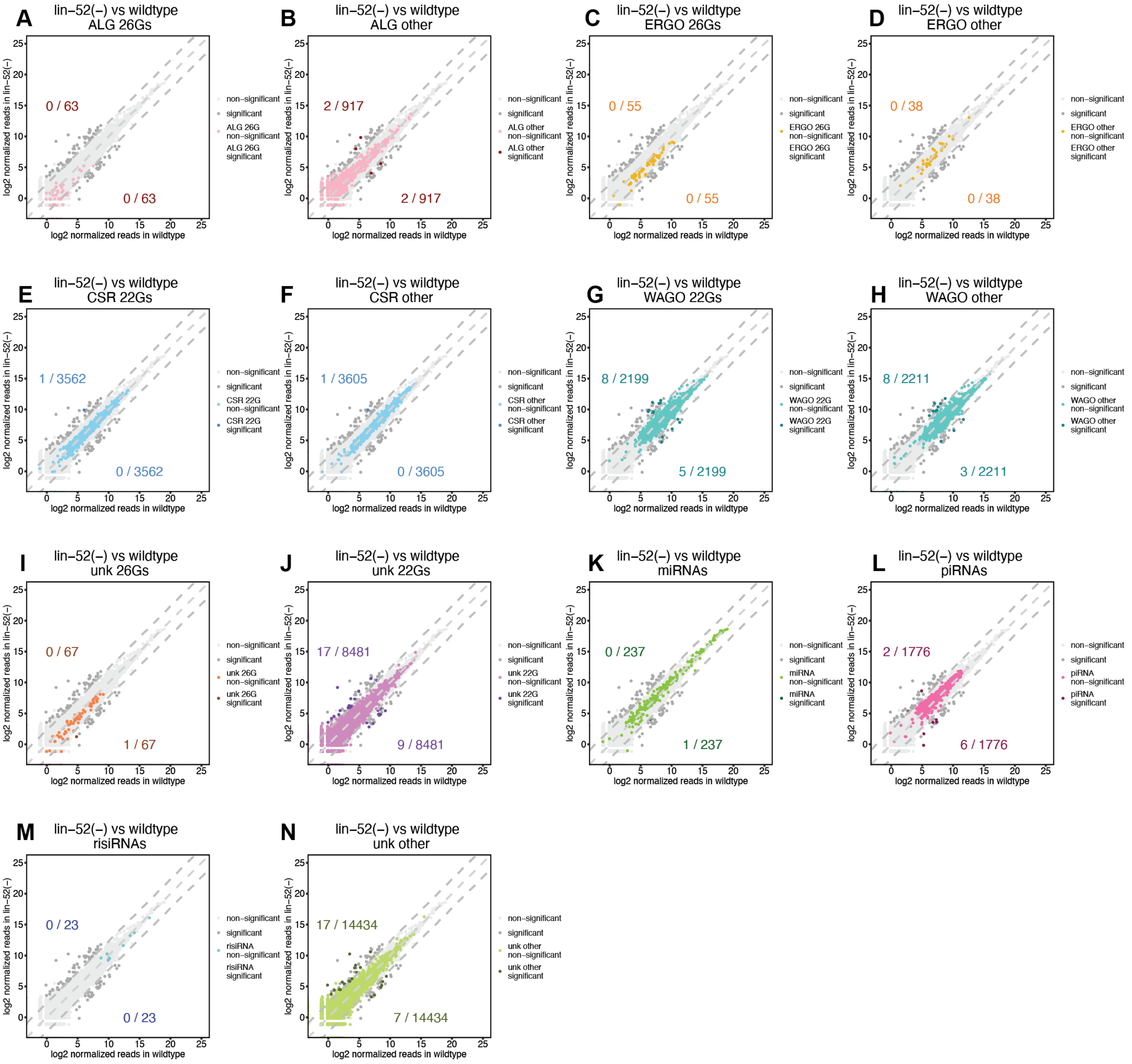

Supplement: 1 [file NIHPP2024.07.12.603258v1-supplement-1.pdf]
